# Correction: Structural and spectroscopic basis of excitation energy transfer in microbial rhodopsins binding xanthophylls

**DOI:** 10.1039/d6sc90054b

**Published:** 2026-02-25

**Authors:** Giacomo Salvadori, Piermarco Saraceno, Alisia Santomieri, Chris John, Laura Pedraza-González

**Affiliations:** a Institute for Computational Biomedicine (INM-9), Forschungszentrum Jülich 52428 Jülich Germany; b Dipartimento di Chimica e Chimica Industriale, Università di Pisa Via G. Moruzzi 13 56124 Pisa Italy laura.pedraza@unipi.it

## Abstract

Correction for “Structural and spectroscopic basis of excitation energy transfer in microbial rhodopsins binding xanthophylls” by Giacomo Salvadori *et al.*, *Chem. Sci.*, 2025, **16**, 18423–18437, https://doi.org/10.1039/D5SC04961J.

The authors regret that an error was present in the construction of the spectral densities employed in the simulations reported in the original article. Specifically, the low-frequency portion of the spectral density for both the retinal and the carotenoid were inadvertently truncated (frequencies <150 cm^−1^ for Kin4B8-lutein and <300 cm^−1^ for Kin4B8-zeaxanthin). This omission affected the vibronic structure of the carotenoid S_2_ state and consequently modified the simulated absorption and circular dichroism (CD) spectra, as well as the spectral overlap entering the Förster excitation energy transfer (EET) rates.

The corrected absorption and CD spectra, previously reported in Fig. 5 in the main text, are shown in the updated Fig. 5 herein.

**Fig. 1:**
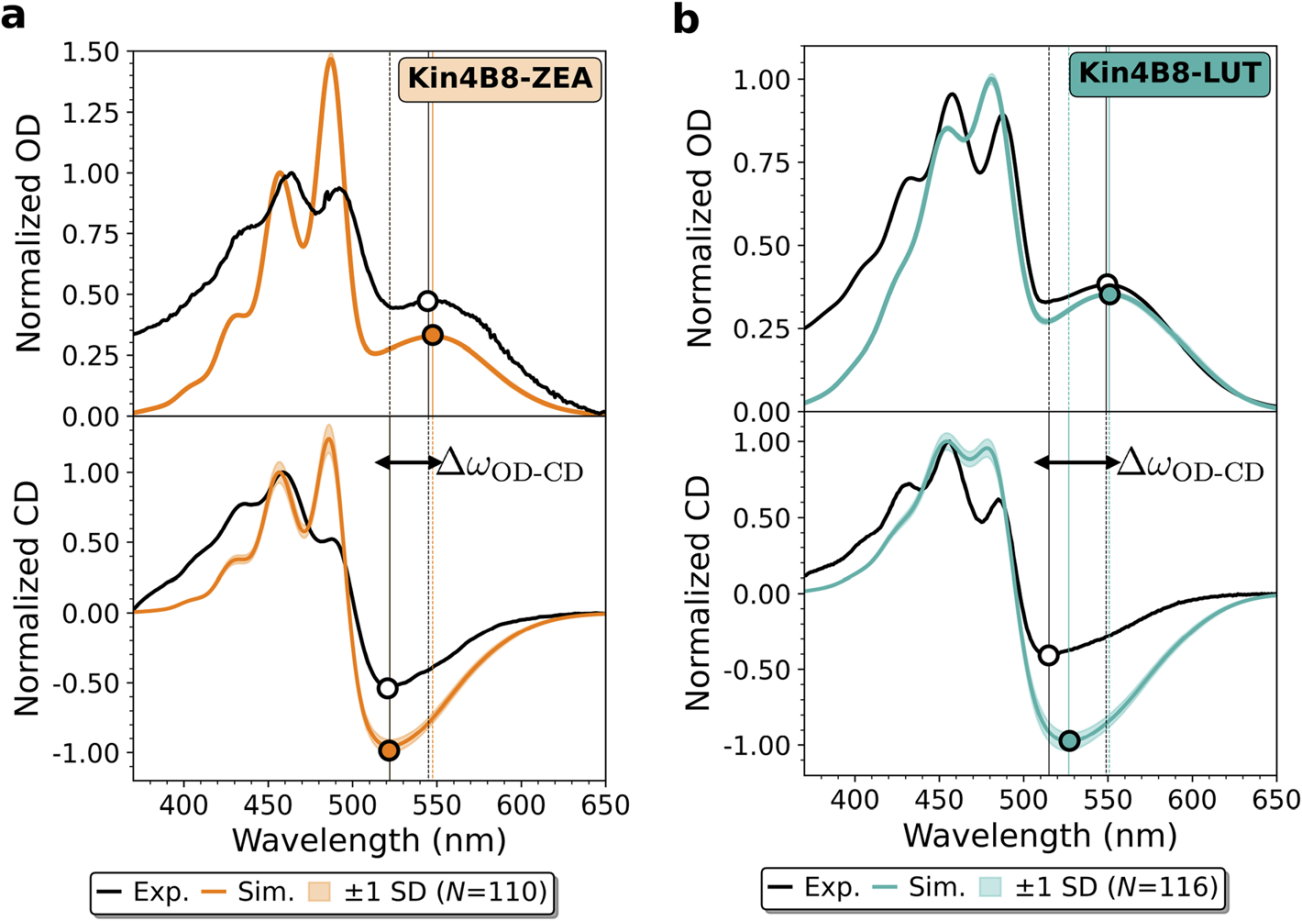
Simulated absorption and circular dichroism spectra of the (a) Kin4B8-ZEA and (b) Kin4B8-LUT complexes. Each spectrum represents the ensemble average of *N* = 110 (ZEA) and *N* = 116 (LUT) replicas. The experimental and calculated spectra are normalized separately to the central carotenoid peak (∼470 nm) for absorption, and to the highest positive peak for CD. Shaded regions indicate the standard deviation estimated *via* bootstrapping over 1500 resampled ensembles. Absorption maxima and CD minima in the *r*PSB region are indicated by circles, as well as by vertical dashed and solid lines, respectively. Experimental spectra are adapted with permission from ref. 18.

The revised absorption spectra display modest but systematic differences relative to those originally published. In the Kin4B8-lutein complex, the relative intensity of the two carotenoid absorption peaks in the 450–500 nm region is altered: whereas the original spectra displayed peaks of comparable height, the corrected spectra exhibit a slightly enhanced intensity for the longer-wavelength peak. In addition, the carotenoid absorption maxima are blue-shifted by approximately 5 nm. A similar trend is observed for the Kin4B8-zeaxanthin complex, where the narrower peaks lead to slightly more pronounced differences. Despite these changes, the overall agreement between simulated and experimental absorption spectra remains good, and all conclusions regarding the reliability of our exciton model are preserved.

The CD spectra are affected in an analogous manner. For Kin4B8-lutein, the carotenoid CD features exhibit a 4–5 nm blue-shift, and the central positive lutein band near 480 nm becomes slightly more intense. The negative CD band at ∼525 nm becomes marginally deeper; however, its characteristic blue-shift relative to the *r*PSB absorption maximum, one of the key spectroscopic signatures of the Kin4B8-xanthophyll complexes, remains essentially unchanged. For Kin4B8-zeaxanthin, the corrections are even less pronounced, and the blue-shift slightly improves the peak positions.

Importantly, correcting the spectral density has a negligible impact on the computed EET times (*τ*_EET_) and efficiencies (*θ*_EET_). For Kin4B8-ZEA, the updated values are *τ*_EET_ = 60.1 ± 1.6 and *θ*_EET_ = 70.1 ± 0.5% (previously 59.1 ± 1.7 and 70.5 ± 0.6%, respectively). For Kin4B8-LUT, the revised values are *τ*_EET_ = 60.7 ± 3.5 and *θ*_EET_ = 70.4 ± 1.2% (previously 60.3 ± 3.5 and 70.5 ± 1.2%, respectively). The updated data are reported in Table S5 in the SI.

Overall, although the corrected spectral densities lead to minor quantitative changes in the simulated optical spectra, all qualitative trends and mechanistic conclusions reported in the original publication remain valid. In particular, the excitonic model continues to reproduce the key experimental observables, including the characteristic shift between the *r*PSB absorption maximum and the negative CD band, as well as the high EET efficiencies observed for both lutein and zeaxanthin.

In addition, in the original versions of Fig. S6b, S7b, S8b, and S9b, a factor of 2 was inadvertently omitted in the excitonic contribution to the CD spectra due to a plotting error. This issue affected only the graphical representation of the data; the underlying calculations were performed correctly. This can be verified by reconstructing the total CD spectrum as the sum of the individual chromophore contributions and the excitonic contribution, which reproduces the reported total spectrum.

The affected figures in the SI have been updated accordingly (Fig. S3 and S6–S10).

The Royal Society of Chemistry apologises for these errors and any consequent inconvenience to authors and readers.

